# Comparison of overall survival and perioperative outcomes of laparoscopic pancreaticoduodenectomy and open pancreaticoduodenectomy for pancreatic ductal adenocarcinoma: a systematic review and meta-analysis

**DOI:** 10.1186/s12885-019-6001-x

**Published:** 2019-08-07

**Authors:** Yu-Li Jiang, Ren-Chao Zhang, Yu-Cheng Zhou

**Affiliations:** 1 School of Medicine, Hang Zhou Normal University, Hangzhou, 310018 Zhejiang Province China; 2 Department of Gastrointestinal and Pancreatic Surgery, Zhejiang Provincial People’s Hospital, Key Laboratory of Gastroenterology of Zhejiang Province, People’s Hospital of Hangzhou Medical College, 158 Shangtang Road, Hangzhou, 310014 Zhejiang Province China

**Keywords:** PDAC, Laparoscopic pancreaticoduodenectomy, Open pancreaticoduodenectomy, Oncological outcome

## Abstract

**Background:**

The aim of this study was to compare the oncological outcomes and clinical efficacy of laparoscopic pancreaticoduodenectomy (LPD) and open pancreaticoduodenectomy (OPD) in patients with pancreatic ductal adenocarcinoma (PDAC).

**Methods:**

We systematically searched PubMed, EMBASE, Web of Science, ClinicalTrials.gov and the Cochrane Central Register for studies published between May 1998 and May 2018. The included studies compared LPD and OPD for the treatment of PDAC. The oncological outcomes and perioperative data were analyzed.

**Results:**

Eight studies involving 15,278 patients were included in our meta-analysis. No significant difference was found in the 5-year overall survival (OS) between patients undergoing the two types of surgery (HR: 0.97, 95% CI 0.82–1.15, *p* = 0.76). LPD resulted in a higher rate of R0 resection than OPD (OR: 1.16, 95% CI 0.85–1.57, *p* > 0.05). This study showed that compared with OPD, LPD resulted in comparable rates of postoperative pancreatic fistulas (POPFs) (OR: 1.07, 95% CI: 0.68–1.68, *p* = 0.77) and postoperative hemorrhage (OR: 1.74, 95% CI 0.96–3.71, *p* = 0.07), more harvested lymph nodes (WMD: 1.84, 95% CI: 0.95–2.72, *p* < 0.05), shorter hospital stays (WMD: -2.45, 95% CI: − 3.33- -1.56, *p* < 0.05), and less estimated blood loss (WMD: -374.30, 95% CI: − 513.06- -235.54, *p* < 0.05).

**Conclusions:**

LPD is equivalent to OPD with respect to 5-year OS and results in better perioperative clinical outcomes for patients with PDAC.

## Background

Pancreaticoduodenectomy (PD) is the main therapy for resectable and borderline resectable pancreatic ductal adenocarcinoma (PDAC) [1]. PD is challenging for surgeons due to the complexities involved in intra-abdominal dissection and the difficulties in reconstructing the alimentary tract; PD has high risks of perioperative morbidity and mortality. Laparoscopic distal pancreatectomy has widely been used to treat benign tumors and is selected to treat adenocarcinoma in the pancreatic tail. In 1994, Ganger performed the first laparoscopic pancreaticoduodenectomy (LPD) [2]. However, over the last decade, LPD has not been universally adopted. The complexity of the procedure, the difficulties involved in anastomoses and postoperative complications may contribute to the unfeasibility of the wide application of this technique. Asbun et al. reported a study demonstrating that LPD could feasibly be performed and could result in shorter hospital stays, less blood loss and better lymph node dissection [3]. In recent years, studies have addressed the efficacy and safety of LPD and OPD. Studies have shown that LPD results in better visual magnification, better exposure, and more delicate manipulation of the deep and difficult to reach tissues.

However, whether the oncological outcomes are equivalent after the application of these two surgical methods is still controversial. Recent studies have reported that LPD can achieve the same oncological outcomes as OPD. Conrad et al. performed a retrospective study involving 65 patients and found that LPD was noninferior to OPD with respect to long-term outcomes for patients with PDAC [4].

However, those seeking to choose between LPD or OPD to treat pancreatic cancer still lack adequate evidence-based medical research. Wang et al. conducted a meta-analysis of 27 studies in 2016 and suggested that microinvasive surgical methods could result in the same clinical outcomes as OPD [4]. That study included various surgical approaches (LPD, robotic pancreaticoduodenectomy (RPD), laparoscopic-assisted pancreaticoduodenectomy (LAPD) and robot-assisted pancreaticoduodenectomy (RAPD)) and different pathologies. Chen et al. performed a meta-analysis in 2018 comparing LPD and OPD for the treatment of pancreatic periampullary cancer and PDAC [5]. No larger, multi-center studies have reported the clinical outcomes of LPD and OPD for only PDAC. The aim of this meta-analysis was to compare the clinical efficacy of LPD and OPD in patients with PDAC with regard to OS.

## Methods

This meta-analysis was performed in accordance with the Preferred Reporting Items for Systemic Reviews and Meta-analysis (PRISMA) statement. We searched PubMed, EMBASE, Web of Science, ClinicalTrials.gov and the Cochrane Central Register for studies published in English between May 1998 and May 2018. The search terms were pancreatic tumor, pancreatic cancer, pancreas ductal adenocarcinoma, PDAC, pancreaticoduodenectomy, PD, laparoscopic pancreaticoduodenectomy, LPD, open pancreaticoduodenectomy, OPD, and LPD and OPD. We also used the combined Boolean operators “AND” or “OR” Title/Abstract. Two investigators reviewed the results together in the case of discrepancies. The inclusion criteria were as follows: (1) comparison of LPD or and OPD for the treatment of PDAC and (2) the evaluation of at least one oncological result, such as recurrence-free survival (RFS), cancer-specific survival (CSS), and OS.

**Fig. 1:**
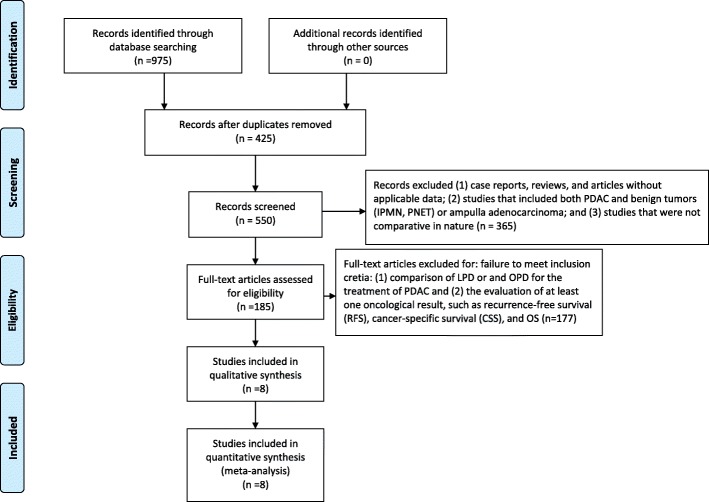
Flow diagram of the process for the selection of relevant studies

The exclusion criteria were as follows: (1) case reports, reviews, and articles without applicable data; (2) studies that included both PDAC and benign tumors (IPMN, PNET) or ampulla adenocarcinoma; and (3) studies that were not comparative in nature. The process of identifying relevant studies is summarized in Fig. 1.

### Statistical analysis

We used Review Manager Version 5.2 (The Cochrane Collaboration, Oxford, UK) to analyze the data. We used the GRADE approach to evaluate the quality of the evidence. We used Cochran’s Q to evaluate the heterogeneity; if the value of Q was< 50% or the *P* value was > 0.01, the heterogeneity was low. However, if the value of Q was > 50% or the *P* value was < 0.01, heterogeneity existed. When I^2^ was > 50%, the random effects model was applied. For quantitative data, we used the weighted mean difference (WMD) or standard mean difference (SMD) for continuous variables. We used odd ratios (ORs) and 95% confidence intervals (CIs) for binary data. We used natural logarithm hazard ratios (LnHRs) and standard error (SE) to pool the 5-year OS data.

## Results

Eight studies were included in our meta-analysis. The process of obtaining these studies is summarized in Fig. 1. From the selected databases, 975 studies were obtained. After screening the titles and abstracts, 425 studies were excluded. After detailed processing of the remaining studies, an additional 417 studies were excluded. Finally, eight studies were included in our meta-analysis [5–12]. Table 1 summarizes the baseline characteristics and assessments of the eight included studies.

**Table 1:** Basic Characteristics of the Included Studies

Study	Year	Design	Sample Size		Mean age(years)		Tumor Size(cm)		BMI (kg/m^2^)		Neoadjuvant therapy (sample size)	
			LPD	OPD	LPD	OPD	LPD	OPD	LPD	OPD	LPD	OPD
Croome	2014	P,S	108	214	66.6	65.4	3.3^a^	3.3^a^	27.4^a^	27.2^a^	12	30
Speicher	2014	R,S	25	84	61	64	2^b^	3^b^	24^b^	25^b^	9	26
Sharpe	2015	R,M	384	4037	65.6	66.1	3.2^a^	3.3^a^	NA	NA	NA	NA
Stauffer	2016	P,S	58	193	66.9	68.9	2.5^b^	3.5^b^	25.9^b^	25.6^b^	NA	NA
Champman	2017	R,M	248	1520	79.6	79.5	NA	NA	NA	NA	175	83
Kantor	2017	R,M	828	7385	65.9	65.7	NA	NA	NA	NA	642	5714
Chen	2018	R,S	47	55	63	66	NA	AN	24^a^	22.7^a^	NA	NA
Dokmak	2015	P,S	46	46	60	63	2.82^a^	2.51^a^	22.6^a^	26.4^a^	NA	NA

*P* prospective study *S* single center *R* retrospective study *M* mutli-centers *NA* not avaliable *BMI* body mass index ^*a*^Mean ^*b*^Median

### Quality assessment

We used the New-Ottawa Scale (NOS) to evaluate the risk of bias in the included studies. The NOS scores were evaluated using a 9-point system. An NOS score of 7 or above is considered to indicate high quality, and an NOS score of 3 or below is considered to indicate low quality. Two reviewers assessed the quality of the included studies. Table 2 shows the risk of bias of the selected studies.

**Table 2:** Newcastle-Ottawa Scale for risk of bias assessment of the included studies

Study	Design	Selection				Comparability	Outcome			Total
		Representativeness of exposed cohort	Selective of nonexposed Cohort	Ascertainment of exposure	Outcome not present at start		Assessment of outcome	Adequate follow-up length	Adequacy of follow-up	
Croome	P	*		*	*	*	*	*	*	7
Speicher	R	*		*	*	*	*	*	*	7
Sharpe	R	*		*	*		*	*	*	6
Stauffer	P	*	*	*	*	*	*	*	*	8
Champman	R	*	*	*	*	*	*	*	*	8
Kantor	R	*	*	*	*	*	*	*	*	8
Chen	R	*	*	*	*	*	*	*	*	8
Dokmak	P	*		*	*	*	*	*	*	7

*P* Prospectively study, *R* Respectively study

### Evidence grading

We used the GRADE approach to evaluate the evidence. This approach includes assessing the underlying quality of the evidence, assessing the major statistical results, and grading the evidence for each outcome. The evidence was categorized as high, moderate, low or very low quality. The criteria for the evaluation of the evidence included the assessment of the risk of bias as determined by the Cochran Risk of Bias Tool [13]. Table 3 shows the summary of the findings of the GRADE approach.

**Table 3:** Summary of findings according to GRADE profiler

Outcome	No. of studies	Study design	Quality assessment					Quality	Importance
			Risk of bias	Inconsistency	Indirectness	Imprecision	Other considerations		
R0 resection	6	Observational studies	Not serious	Not serious	Not serious	Not serious	Not serious	Low	Critical
POPF	5	Observational studies	Not serious	Not serious	Not serious	Not serious	Not serious	Low	Critical
Lymph node harvested	4	Observational studies	Not serious	Not serious	Not serious	Not serious	Not serious	Low	Critical
Postoperative Hemorrhage	4	Observational studies	Not serious	Not serious	Not serious	Not serious	Not serious	Low	Critical
Estimated Blood loss	4	Observational studies	Not serious	Not serious	Not serious	Not serious	Not serious	Low	Critical
Length hospital days	5	Observational studies	Not serious	Not serious	Not serious	Not serious	Not serious	Low	Critical

### 5-year overall survival

**Fig. 2:**
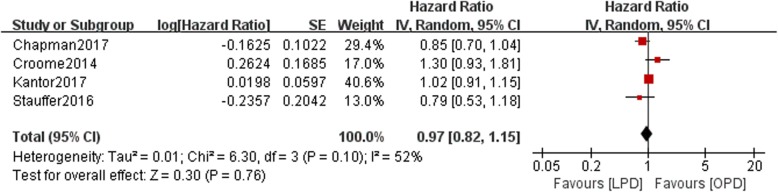
Forest plot for 5-year OS between the LPD and OPD for PDCA

Data on 5-year OS were available in four studies. There was a statistically significant difference in OS between the LPD and OPD groups with moderate heterogeneity (*n* = 10,554, 1242 patients were in the LPD group, 9312 patients were in the OPD group, HR: 0.97, 95% CI 0.82–1.15, *p* = 0.76, I^2^ = 52%, random-effective model, Fig. 2).

### R0 resection

**Fig. 3:**
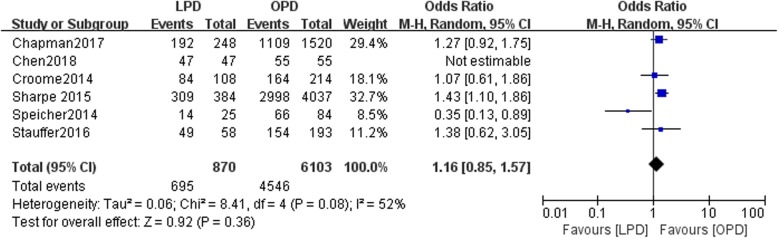
Forest plot for R0 resection between the LPD and OPD for PDCA

Six studies included data on R0 resection. There was a statistically significant difference in R0 resection between the LPD and OPD groups with moderate heterogeneity (*n* = 6973, 870 patients were in the LPD group, and 6103 patients were in the OPD group, OR: 1.16, 95% CI 0.85–1.57, *p* = 0.36, I^2^ = 52%, random-effective model, Fig. 3).

### Harvested lymph nodes

**Fig. 4:**
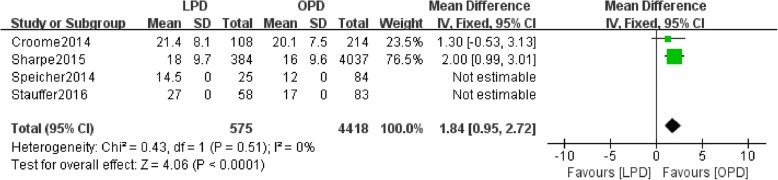
Forest plot for Lymph node harvested between the LPD and OPD for PDCA

Data regarding the number of harvested lymph nodes were available in four studies. There was a statistically significant difference in the number of harvested lymph nodes between the LPD and OPD groups (*n* = 4993, 575 patients were in the LPD group, and 4418 patients were in the OPD group, WMD: 1.84, 95% CI 0.95–2.72, *p* < 0.0001, I^2^ = 0, fixed effects model, Fig. 4).

### Postoperative pancreatic fistula

**Fig. 5:**
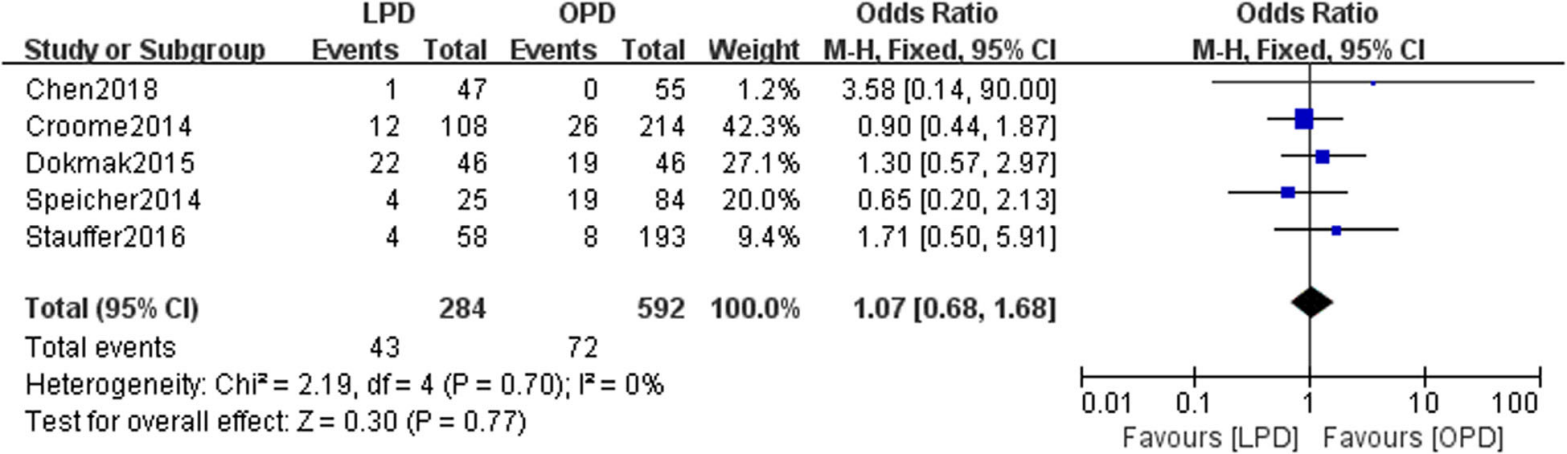
Forest plot for POPF between the LPD and OPD for PDCA

Five studies reported the incidence of postoperative pancreatic fistulas (POPFs) in the LPD and OPD groups. No statistically significant difference in the incidence of POPFs existed between the two groups (*n* = 876, 284 patients were in the LPD group, and 592 patients were in the OPD group, OR: 1.07, 95% CI 0.68–1.68, *p* = 0.77, I^2^ = 0, fixed effects model, Fig. 5).

### Estimated blood loss

**Fig. 6:**
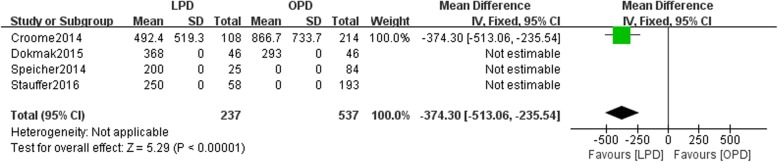
Forest plot for Estimated blood loss between the LPD and OPD for PDCA

Four studies included data regarding the estimated blood loss (ESBL). There was a statistically significant difference in ESBL between the LPD and OPD groups (*n* = 774, 237 patients were in the LPD group, and 537 patients were in the OPD group, WMD: -374.30, 95% CI -513.06--235.54, *p* < 0.05, fixed effects model, Fig. 6).

### Postoperative bleeding

**Fig. 7:**
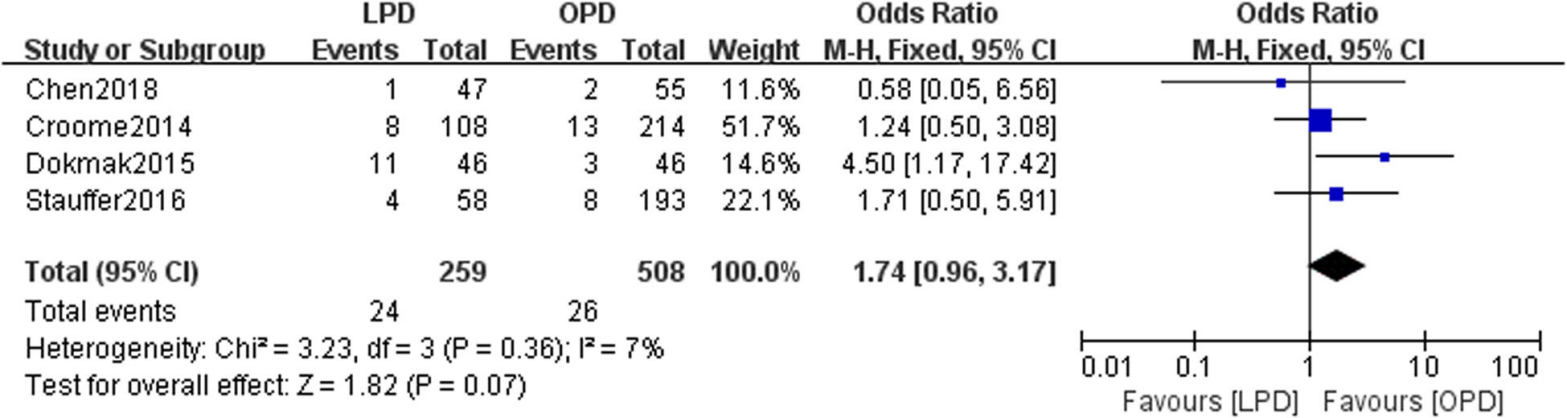
Forest plot for Postoperative bleeding between the LPD and OPD for PDCA

Four studies reported postoperative bleeding in the LPD and OPD groups. No statistically significant difference in postoperative bleeding existed between the two groups (*n* = 767, 259 patients were in the LPD group, and 508 patients were in the OPD group, RR: 1.74, 95% CI 0.96–3.17, *p* = 0.07, I^2^ = 7%, fixed effects model, Fig. 7).

### Hospital stay

**Fig. 8:**
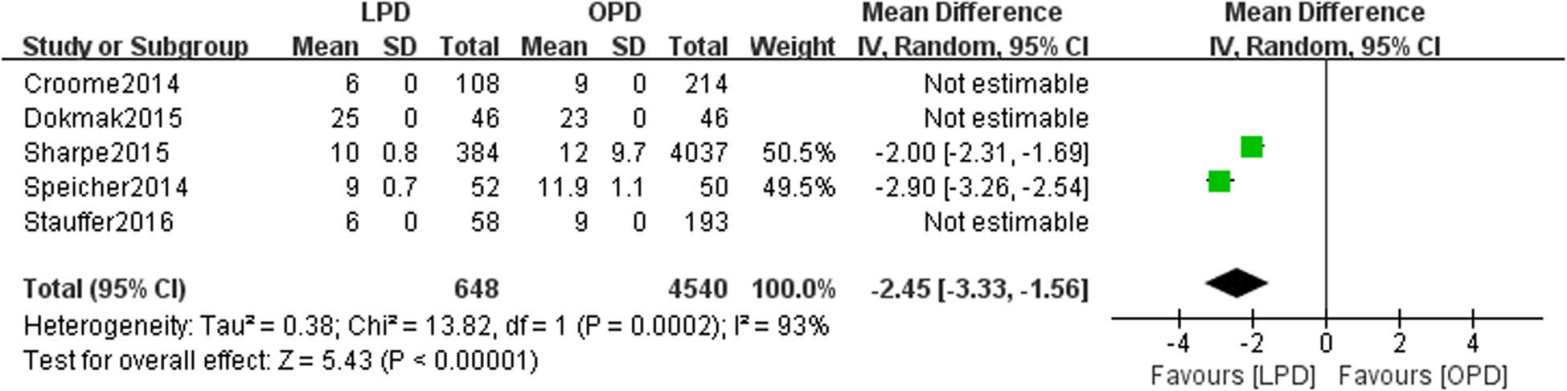
Forest plot for Hospital stay between the LPD and OPD for PDCA

Data on the duration of hospital stays were available in five studies. There was a statistically significant difference in hospital stay duration between the LPD and OPD groups with high heterogeneity(*n* = 5188, 648 patients were in the LPD group, 4540 patients were in the OPD group, WMD: -2.45, 95% CI -3.33- -1.56, *p* < 0.05, I^2^ = 93%, random-effective model, Fig. 8).

## Discussion

This meta-analysis is the first study to evaluate clinical efficacy of LPD and OPD for the treatment of PDAC with OS as the oncological outcome. We pooled eight studies to compare 5-year OS rates and perioperative clinical outcomes. Our meta-analysis suggested that no significant difference was found in the 5-year OS and POPF rates between the LPD and OPD groups. The incidence of R0 resection, number of lymph nodes harvested, amount of postoperative bleeding and duration of hospital stay were better in the LPD group than in the OPD group. We used the GRADE approach to evaluate the quality of the evidence. This study suggested that compared with OPD, LPD could produce equivalent oncological outcomes and better perioperative outcomes in patients with PDAC. Our study report represents the largest study to date comparing 5-year OS rates for patients with PDAC treated with LPD or OPD.

We find that LPD is equivalent to OPD in terms of the oncological outcome, the 5-year OS rate (*p* = 0.76) (Fig. 2). However, Chapaman et al. performed a study with 1668 patients aged > 75 years identified by searching the NCDB. They found that after adjusting for common factors, the LPD group achieved a better OS rate than the OPD group (HR 0.85, 95% CI 0.69–1.03) [7]. This was consistent with the findings of our study (HR 0.97, 95% CI 0.82–1.15) (Fig. 2). Kantor et al. found that the 5-year OS rate was longer in the LPD group than in the OPD group (*p* = 0.68), although the difference was not significant. They found that chemotherapy or radiation therapy may predict the OS rate according to the Cox regression results [6]. However, Stauffer et al. conducted a study and found no differences in patient demographics and 5-year OS rates between the two groups [10]. They found that the rates of postoperative complications were comparable between the two groups. Furthermore, they concluded that major postoperative complications were independent factors associated with the 5-year OS rate [10]. This finding may be related to the time interval between surgery and postoperative chemotherapy or adjuvant chemotherapy, as reported by Nussbaum et al. in 2016 [14]. Similarly, Chen et al. performed a meta-analysis comparing periampulla cancer and PDAC. They conducted a subgroup analysis of patients with PDAC using relative risk (RR) instead of HRs to pool the 5-year overall survival and found similar results to those of our study [15].

The present study showed that compared with OPD, LPD resulted in a higher rate of R0 resection (*P* = 0.009) (Fig. 3). Conversely, Croome et al. performed a study with 322 patients (108 patients in the LPD group and 214 patients in the OPD group). In the study by Croome et al., no statistically significant difference was found in the rates of R0 resection between the two groups (*p* = 0.81) [8].

In a study similar to ours with 251 patients (193 patients in the OPD group, 58 patients in the LPD group), Stauffer et al. found that LPD and OPD may result in different margin statuses according to both multivariate and univariate analyses (*p* = 0.025, *p* < 0.001 respectively) [10].

They performed a Cox proportional hazards regression analysis and found that the presence of positive margins (R1/R2) and lymph node ratio could predict the 5-year OS rate (*p* = 0025, *p* < 0.001 respectively). However, in the multivariate analysis, Croome et al. found that only positive nodal status (HR = 1.7; 95% CI, 0.94–3.2; *P* = 0.076) was associated with the 5-year OS rate. However, this study may include bias because the patients had different types of pancreatic malignancies. Delitto and colleagues performed a retrospective study involving 236 PDs, with 102 patients (52 patients in the LPD group and 50 patients in OPD group) with periampullary malignancies [16]. They found that the R1 resection (HR = 1.20; 95%CI, 0.53–2.72; *P* = 0.663) predicted no significant correlation with overall survival of LPD and OPD in Univariate analysis. Zureikat et al. reported a study involving 28 patients, with 14 patients in the LPD group and 14 patients in the OPD group. No difference was found in the rate of R0 resection between the two groups (LPD 100% vs. OPD 91.7%, *P* = 0.31) [17]. This study involved different histological types (benign and malignant), which might reduce the quality of the evidence.

In the present study, LPD resulted in a greater number of harvested lymph nodes than OPD group (*P* < 0.05) Fig. 4. In a recent study, Stauffer et al. performed a study that included 251 patients (193 patients in the OPD group and 58 patients in LPD group) and found that OPD resulted in more harvested lymph nodes than LPD (*P* < 0.01) [10]. They found that the numbers of harvested lymph nodes were not significantly different between the two groups [10], which is similar to our result. However, Croome et al. conducted a retrocpective study and found no statistically significant difference in the number of harvested lymph nodes between the LPD and OPD groups (*P* = 0.15) [8]. Recent studies have also used the lymph node ratio (LNR) to assess and stage PDAC. Zhang et al. performed a retrospective study of 83 patients. After performing univariate and multivariate analyses, they found that a LNR > 0.2 could be an independent predictor of OS (*p* = 0.018, HR = 2.865) [18].

Regarding the occurrence of POPFs, our meta-analysis indicated that no statistically significant difference was found between the two groups (*P* > 0.05). However, Croome et al. and Specicher et al. found that the rate of POPFs might be higher in the LPD group than in the OPD group [8, 9]. POPFs are serious complications that are associated with the reconstructive skills of the surgeon. Similar to our results, Stauffer et al. found that compared with the LPD group, the OPD group had a higher rate of POPFs (OPD 12.3%, LPD 11.8%, respectively), although the difference was not significant (*p* = 0.912). Additionally, Chen conducted a meta-analysis of studies that investigated patients with PDAC and periampullary cancer and found no difference in the rates of POPFs between the LPD and OPD groups in a subgroup analysis (RR 0.90 95% CI 0.52–1.56, *p* = 0.70) [15]. Similarly, Delitto et al. reported that compared with the OPD group, the LPD group had a lower POPF rate (*p* = 0.032) [16]. A lower rate of POPFs in the LPD group than in the OPD group may be explained by the improved visibility of the deeper abdominal structure during LPD. However, their study included different pathologies (PDAC, ampullary adenocarcinoma, cholangiocarcinoma, and duodenal adenocarcinoma). Therefore, the finding may be due to selection bias. In addition, it may be related to the greater familiarity of surgeons in high-volume centers than of surgeons in low-volume centers with the skills involved in LPD. According to previous publications regarding POPFs, the reported incidence of POPFs after OPD ranges from 2.0 to 36.0%, while the reported incidence after LPD ranges from 0 to 35.0% [17, 18]. Chen et al. also found comparable POPF rates after LPD and OPD (12.8% vs. 14.5%; *P* = 0.67), which was similar to the result we found in our study [5].

In our meta-analysis, the rate of postoperative bleeding was not different between the LPD and OPD groups. Chen et al. reported that compared with the OPD group, the LPD group had less postoperative bleeding. However, Cromme et al. and Stauffer et al. found that a higher rate of postoperative bleeding after LPD than after OPD. Increased postoperative bleeding may be associated with a higher rate of conversion or shorter overall survival.

In our study, the hospital stay length was not significantly different between the LPD and OPD groups. Several studies have reported similar results. This finding may not be related to the 5-year OS rates. In this study, the relationship between the LPD and OPD groups was not confirmed.

The ESBL in our study was smaller in the LPD group than in the OPD group (Fig. 6). Nagakawa et al. performed a cohort study that involved 42 patients (21 in the left superior mesenteric artery (SMA) group and 21 in the right SMA group). They suggested that the right side of the SMA facilitates dissection of the inferior pancreaticoduodenal artery (IPDA), thereby reducing operative times. Stauffer et al. reported that a lower LNR was associated with less blood loss and a lower complication rate [10]. Nagakawa indicated that bleeding from the jejunal vein and inferior pancreatico-duodenal vessels (IPDVs) often occurred during dissection of the left side of the SMA [10]. An adequate surgical window and greater visibility during LPD than during OPD ensure easier control of the root of the IPDA. These findings indicate that removing additional peripancreatic lymph nodes may be associated with less blood loss.

In our meta-analysis, data from eight published studies comparing LPD and OPD for the treatment of PDAC were pooled in this study. Two studies (Sharpe2015, Kantor2016) used data from the NCDB to compare the two methods, which may have resulted in selection bias. One study (Chapman2017) focused on elderly patients, and one study used a propensity score matching analysis. After pooling the studies, heterogeneity existed. Additionally, the included studies had small sample sizes.

Our study also had some limitations. First, not all the included studies reported the 5-year OS and other oncological outcomes. This does impact the quality of the evidence. Second, the included studies involved different centers with surgeons who had different surgical skill levels, which does result in bias. Third, there was not a consensus regarding the definition of a positive margin. No RCTs were included in this meta-analysis. This does impact the level of evidence. The different common base characteristics does increase the heterogeneity. For example, the tumor size, neoadjuvant therapy and BMI are the covariates to impact the outcomes (oncological, mortality and morbidity) in PDAC. Besides, we could not eliminate the selection bias (surgeons use LPD or OPD according to their preference). The higher cost of LPD compared with OPD is a critical issue. However, the included studies did not perform cost analyses. More RCTs and data including use of neoadjuvant, histology, tumor size, differentiation, BMI and stented or not are pooled to verify overall survival and perioperative outcomes of laparoscopic pancreaticoduodenectomy and open pancreaticoduodenectomy for PDAC.

## Conclusions

In our meta-analysis, LPD and OPD achieved equivalent 5-year OS rates for the treatment of PDAC. Compared with OPD, LPD resulted in a higher rate of R0 resection, a greater number of harvested lymph nodes, shorter hospital stays, a lower rate of postoperative hemorrhage, less ESBL, and an equivalent rate of POPFs. LPD may be superior to OPD for patients with PDAC.

## Data Availability

All data generated or analyzed during this study are included in this published article.

## Abbreviations

CI: Confidence interval
CSS: Cancer-specific survival
ESBL: Estimated blood loss
HR: Hazard ratio
IPDVs: Inferior pancreatico-duodenal vessels
LNR: Lymph node ratio
LPD: Laparoscopic pancreaticoduodenectomy
NCDB: National Cancer Data Base
OPD: Open pancreaticoduodenectomy
OR: Odds ratio
OS: Overall survival
PD: Pancreaticoduodenectomy
PDAC: Pancreatic ductal adenocarcinoma
POPF: Postoperative pancreatic fistula
RFS: Recurrence-free survival
SMA: Superior mesenteric artery
WMD: Weight mean difference
